# Income and Geographic Disparities in in‐Hospital Mortality Following Cardiovascular Procedures: Evidence From the National Inpatient Sample, 2016–2022

**DOI:** 10.1111/1475-6773.70117

**Published:** 2026-04-16

**Authors:** Alon Bergman, Ashwin Nathan

**Affiliations:** ^1^ Department of Medical Ethics and Health Policy, Perelman School of Medicine University of Pennsylvania Philadelphia Pennsylvania USA; ^2^ Department of Health Care Management, the Wharton School University of Pennsylvania Philadelphia Pennsylvania USA; ^3^ Division of Cardiology, Department of Medicine University of Pennsylvania Philadelphia Pennsylvania USA

**Keywords:** cardiovascular diseases, disparities, geography, mortality, socioeconomic factors

## Abstract

**Objective:**

To examine whether income‐ and geography‐related disparities in in‐hospital mortality after major cardiovascular procedures arise from differences in patient acuity, hospital characteristics, or inequities within hospitals.

**Study Setting and Design:**

This observational study analyzed national data on eight major cardiovascular procedures performed between 2016 and 2022. We used multivariable logistic regression with progressive adjustment for demographics, clinical severity (All Patient Refined Diagnosis Related Groups [APR‐DRG] risk and severity scores), and hospital characteristics.

**Data Sources and Analytic Sample:**

We analyzed secondary data from the National Inpatient Sample including 1,120,235 discharges (weighted *N* = 5,906,795) representing adults undergoing percutaneous coronary intervention, coronary artery bypass grafting, carotid endarterectomy/stenting, surgical valve replacement, transcatheter valve procedures, non‐carotid endarterectomy, aneurysm repair, or peripheral bypass. Patient income was proxied using ZIP code‐level median household income quartiles. Geographic location was classified as large metropolitan (≥ 1 million population), smaller metropolitan (50,000–999,999), or non‐metropolitan.

**Principal Findings:**

Lowest‐income patients presented with mean APR‐DRG risk scores 0.15–0.25 points higher than highest‐income patients. After full adjustment with hospital fixed effects, in‐hospital mortality was 0.67% points higher (95% CI: 0.08–1.26) among lowest‐income patients. Geographic patterns were complex: after adjusting for hospital characteristics, non‐metropolitan location was associated with 0.48% points higher mortality, though this was not statistically significant (95% CI: −0.01 to 0.97), and smaller metropolitan areas with 1.03% points higher mortality (95% CI: 0.30–1.76). Between‐hospital differences explained 11.6% of mortality variance.

**Conclusions:**

Socioeconomic and geographic disparities in mortality following major cardiovascular procedures persist after adjustment for clinical and hospital factors. These disparities remain, with slightly larger point estimates, in within‐hospital analyses, suggesting that hospital‐level differences alone do not account for observed inequities. Interventions should address both social determinants and intra‐hospital inequities. Multilevel interventions targeting both social determinants and within‐hospital processes may be needed.

## Summary

- What is known on this topic
  ○ Lower‐income patients and rural residents experience worse cardiovascular outcomes, but whether this reflects patient health or hospital quality remains unclear.
  ○ Previous studies could not determine if disparities stem from which hospitals patients access or from differences in care within individual hospitals
  ○ Performance‐based payment programs may unintentionally penalize hospitals serving disadvantaged populations, potentially worsening existing disparities.
- What this study found
  ○ Income disparities persist even among patients treated at the same hospital, indicating that simply accessing better hospitals cannot eliminate these gaps.
  ○ Geographic disparities largely reflect hospital‐level differences, with smaller and non‐metropolitan facilities having higher mortality rates after cardiovascular procedures.
  ○ Reducing disparities requires both strengthening hospitals in underserved areas and addressing inequitable care patterns for low‐income patients within all facilities.

## 1 Introduction

Cardiovascular disease remains the leading cause of death in the United States, accounting for nearly one in every three deaths annually [1]. After decades of steady decline, progress in reducing cardiovascular mortality has plateaued, and in some groups reversed, since the early 2010s [2, 3]. These trends raise important questions about equity in prevention and treatment and suggest that structural barriers may be limiting the reach of cardiovascular advances.

Substantial evidence demonstrates that cardiovascular outcomes vary systematically by socioeconomic status and geography. Individuals from lower‐income communities experience higher rates of acute myocardial infarction, heart failure, and stroke, with poorer short‐ and long‐term survival [4, 5, 6, 7, 8, 9]. Geographic disparities are similarly pronounced, with rural patients and those treated in smaller hospitals often experiencing worse outcomes than their urban counterparts [10, 11, 12]. Importantly, these inequities are not explained solely by differences in comorbidity or access to specialty care; rather, they reflect structural and systemic determinants of health that shape risk profiles, care processes, and outcomes [13, 14, 15].

Disparities are evident across major cardiovascular procedures. Studies have shown that lower socioeconomic position is associated with worse survival after coronary artery bypass grafting and valve surgery [16, 17], reduced access to advanced therapies such as transcatheter aortic valve replacement [18, 19], and substantial geographic inequality in procedure availability particularly for minimally invasive interventions [20]. Evidence suggests that such disparities persist even among patients treated in the same hospitals, highlighting the need to consider within‐hospital inequities in addition to differences across institutions [21, 22, 23].

These disparities intersect with ongoing U.S. health policy reforms. Programs such as the Hospital Readmissions Reduction Program and value‐based purchasing were designed to improve quality and efficiency but may disproportionately penalize safety‐net hospitals serving vulnerable populations [24, 25, 26, 27]. As a result, disadvantaged patients may face a double burden: higher baseline clinical risk and hospitals constrained by financial penalties. Understanding whether disparities in cardiovascular outcomes stem from hospital‐level factors or persist within institutions is critical to shaping policies that promote equity rather than widen gaps.

Against this backdrop, we used the National Inpatient Sample and All Patient Refined Diagnosis‐Related Groups (APR‐DRG) severity adjustment methods to examine income‐ and geography‐related disparities in in‐hospital mortality across eight major cardiovascular procedures [28, 29]. Our goal was to determine whether disparities reflect differences in patient acuity, hospital characteristics, or inequities that persist within hospitals, with implications for both clinical practice and health policy.

## 2 Methods

### 2.1 Data Source and Study Population

We analyzed data from the National Inpatient Sample (NIS), part of the Healthcare Cost and Utilization Project (HCUP) sponsored by the Agency for Healthcare Research and Quality [29]. The NIS is the largest publicly available all‐payer inpatient healthcare database in the United States, representing approximately 20% of all US hospital discharges from participating community hospitals. The NIS employs a stratified probability design to ensure representation across geographic regions, urban–rural location, teaching status, bed size, and ownership categories. We used data from 2016 to 2022.

Our study included adult patients (age ≥ 18 years) who underwent one of eight major cardiovascular procedures. Procedures were identified using ICD‐10‐PCS codes aggregated by the Procedure Classifications Software Refined for ICD‐10‐PCS (PRCCSR) categories developed by HCUP [30]. We initially identified all procedures within the cardiovascular domain, then selected the eight highest‐volume procedure groups performed in the inpatient setting. We excluded PRCCSR categories not primarily performed in the inpatient setting (pacemaker and defibrillator procedures; angioplasty and related endovascular procedures excluding carotid) as well as those with heterogeneous or poorly defined procedural content (venous and arterial catheter placement; saphenous vein harvest and other therapeutic vessel removal; placement of tunneled or implantable vascular access devices; cardiovascular device procedures not elsewhere classified; vessel repair and replacement; and ligation and embolization of vessels).

The final eight procedure groups were: percutaneous coronary intervention (PCI; PRCCSR 4), coronary artery bypass grafting (CABG; PRCCSR 3), carotid endarterectomy or stenting (CEA/CAS; PRCCSR 6), surgical valve replacement (PRCCSR 22), transcatheter valve procedures including transcatheter aortic and mitral valve replacement (TAVR/TMVR; PRCCSR 23), non‐carotid endarterectomy procedures (Non‐Carotid EA; PRCCSR 7), aneurysm repair (PRCCSR 11), and peripheral bypass (PRCCSR 14). The final analytic sample comprised 1,120,235 discharges (weighted *N* = 5,906,795).

### 2.2 Primary Outcome

The primary outcome was in‐hospital mortality, defined as death during the index hospitalization for the cardiovascular procedure. This outcome was identified using the disposition field in the NIS database.

### 2.3 Exposures of Interest

#### 2.3.1 Patient Income Quartile

We used median household income for the patient's residential ZIP code, categorized into national quartiles based on Census data, as a proxy for individual socioeconomic status (quartile 1 = lowest income areas; quartile 4 = highest income areas).

#### 2.3.2 Patient Location Urban–Rural Setting

**FIGURE 1:**
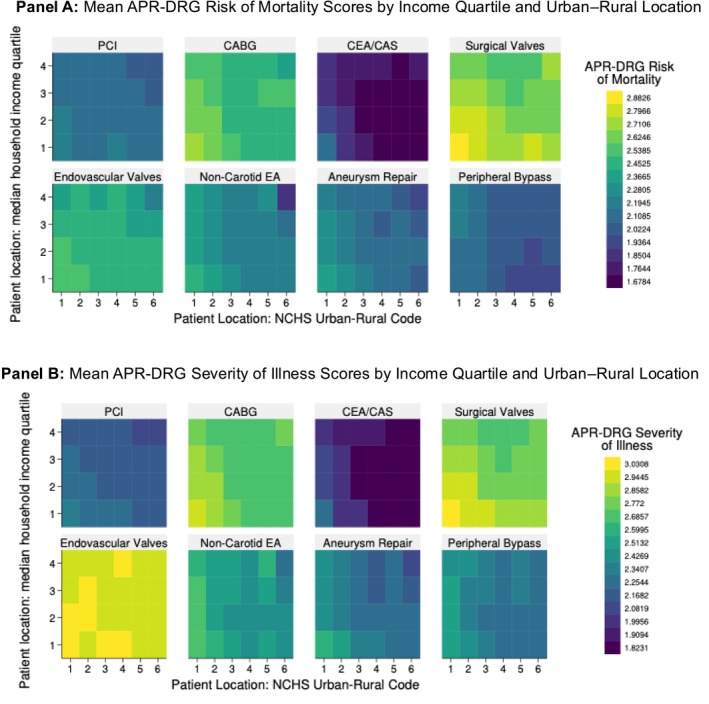
Baseline patient acuity by income quartile and urban–rural classification. Heatmap displaying mean all patient refined diagnosis related groups (APR‐DRG) risk of mortality scores (A) and severity of illness scores (B) across strata defined by patient residential location and income. *X*‐axis shows the full National Center for Health Statistics Urban–Rural Classification Scheme (NCHS) urban–rural classification: 1 = large central metropolitan, 2 = large fringe metropolitan, 3 = medium metropolitan, 4 = small metropolitan, 5 = micropolitan, 6 = noncore (rural). *Y*‐axis shows median household income quartiles (1 = lowest, 4 = highest). Cell shading intensity represents mean score value, with lighter shading indicating higher clinical acuity.

Geographic location was classified using the NIS urban–rural classification based on the National Center for Health Statistics (NCHS) Urban–Rural Classification Scheme. Patient location was categorized into three groups: large metropolitan areas (central counties of metropolitan areas with ≥ 1 million population; NCHS codes 1–2), smaller metropolitan areas (counties in metropolitan areas with 50,000–999,999 population; NCHS codes 3–4), and non‐metropolitan areas (micropolitan and non‐core areas; NCHS codes 5–6). For descriptive heatmap visualizations (Figure 1), we used the complete six‐level NCHS classification: large central metropolitan (codes 1), large fringe metropolitan (2), medium metropolitan (3), small metropolitan (4), micropolitan (5), and noncore/rural (6).

### 2.4 Clinical Severity Measures

We used APR‐DRG risk of mortality and severity of illness scores as measures of baseline clinical acuity. The APR‐DRG system assigns each hospitalization a risk of mortality score (likelihood of dying) and severity of illness score (extent of physiologic decompensation or organ system loss of function) ranging from 1 (minor) to 4 (extreme) [28]. These scores incorporate patient age, principal diagnosis, secondary diagnoses, and procedures performed.

### 2.5 Covariates

Demographic adjustments in Models 1–4 included continuous age, sex, race and ethnicity, and calendar year. Race and ethnicity were obtained from the NIS database, which derives this information from hospital discharge records. Hospitals report race and ethnicity using categories aligned with federal standards; specific methods of data collection vary across hospitals and are not identifiable in the NIS. Categories included Asian or Pacific Islander, Black, Hispanic, White, and Other (American Indian/Alaska Native, individuals reporting multiple races, and those with unknown or missing race and ethnicity data). We included race and ethnicity as covariates as prior research demonstrates significant racial and ethnic disparities in cardiovascular outcomes, and our study aimed to describe the demographic characteristics of patients undergoing these procedures and to adjust for potential confounding by race and ethnicity when estimating income‐ and geography‐related disparities. We recognize that race and ethnicity are social constructs and serve as proxies for exposure to structural racism and differential access to resources rather than reflecting inherent biological differences. To flexibly capture demographic differences in mortality risk, we included all two‐way and three‐way interactions among age, sex, and race and ethnicity. Clinical adjustments in Models 2–4 included APR‐DRG risk of mortality and severity of illness scores, each entered as four‐level categorical variables (minor, moderate, major, extreme).

### 2.6 Statistical Analysis

We calculated descriptive statistics for patient demographics, clinical characteristics, and in‐hospital mortality rates across the eight procedure types. Continuous variables are presented as means with standard deviations; categorical variables as frequencies and percentages.

To examine the relationship between income quartile, geographic location, and in‐hospital mortality, we constructed a series of nested multivariable logistic regression models with progressively more detailed adjustment. In all models, patient income quartile and urban–rural location were included simultaneously as covariates, allowing us to estimate the independent associations of each exposure with mortality while adjusting for the other:

**Model 1:** Adjusted for demographic characteristics including continuous age, sex, race and ethnicity (White, Black, Hispanic, Asian/Pacific Islander, Other), and their two‐way and three‐way interactions (age‐by‐sex, age‐by‐race and ethnicity, sex‐by‐race and ethnicity, and age‐by‐sex‐by‐race and ethnicity), plus year of hospitalization.

**Model 2:** Model 1 plus APR‐DRG risk of mortality and severity of illness scores (each entered as categorical variables with levels 1–4).

**Model 3:** Model 2 specifications fitted using multilevel mixed‐effects logistic regression with hospital random intercepts to account for clustering of patients within hospitals.

**Model 4:** Model 2 specifications fitted using conditional fixed‐effects logistic regression, which compares patients treated at the same hospital by including hospital‐specific intercepts.

This progressive modeling strategy allowed us to assess the magnitude of observed disparities after accounting for demographic differences (Model 1), how much could be explained by patient case‐mix and clinical severity (Model 2), the contribution of between‐hospital variation (Model 3), and whether disparities persisted within individual hospitals (Model 4).

We report average marginal effects with 95% confidence intervals (CIs) for all models. The reference categories were the highest income quartile (quartile 4) and large metropolitan areas (NCHS codes 1–2). For Model 3, we calculated the intraclass correlation coefficient (ICC) to quantify the proportion of variance in mortality attributable to between‐hospital differences.

To examine procedure‐specific disparities, we estimated Model 2 separately for each of the eight cardiovascular procedures and generated forest plots displaying average marginal effects and 95% confidence intervals for income quartiles and patient location categories across procedures.

Models 1–3 accounted for the complex survey design of the NIS using discharge‐level probability weights and variance estimation, adjusting for clustering of patients within hospitals. Model 4 was estimated without survey weights due to the methodological constraints of conditional fixed‐effects logistic regression. Hospitals with fewer than 10 procedure‐specific discharges were excluded from procedure‐specific analyses to ensure stability of estimates. Statistical analyses were performed using Stata version 19. A two‐sided *p* < 0.05 was considered statistically significant.

### 2.7 Ethical Considerations

Because this study used de‐identified publicly available data, it was exempt from institutional review board approval under 45 CFR 46.104(d) (4).

## 3 Results

### 3.1 Patient Characteristics

The study included 1,120,235 cardiovascular procedure hospitalizations from 2016 to 2022, representing an estimated 5,906,795 procedures nationally. Table 1 presents baseline characteristics stratified by procedure type. PCI was the most common procedure (weighted *N* = 2,369,272), followed by CABG (*N* = 975,287) and CEA/CAS (*N* = 563,899). The majority of procedures were performed in large metropolitan areas (range: 45.3%–52.1% across procedure types).

**TABLE 1:** Baseline characteristics of cardiovascular procedures in the NIS (2016–2022).

	PCI	CABG	CEA/CAS	Surgical valves	TAVR/TMVR	Non‐carotid vascular	Aneurysm repair	Peripheral bypass
Unweighted *N* (discharges)	447,646	184,269	106,542	101,067	73,225	73,132	72,264	57,874
Weighted *N* (national)	2,369,272	975,287	563,899	534,921	387,561	387,068	382,474	306,312
Elective admission (%)	9.2	48.6	70.1	69.5	77.7	47.0	57.1	61.5
Mean APR‐DRG risk of mortality	2.12	2.53	1.76	2.69	2.45	2.27	2.16	2.05
	(1.05)	(0.96)	(0.94)	(0.95)	(0.84)	(1.04)	(1.13)	(0.99)
Mean APR‐DRG severity of illness	2.20	2.74	1.93	2.83	2.98	2.53	2.33	2.36
	(1.00)	(0.81)	(0.94)	(0.89)	(1.05)	(0.98)	(1.07)	(0.94)
In‐hospital mortality (%)	2.9	2.4	1.7	3.7	2.1	4.6	4.6	2.9
Mean age (years)	65.4	66.3	69.7	61.1	77.0	65.4	65.9	65.2
	(12.4)	(10.1)	(11.4)	(18.2)	(13.2)	(14.4)	(16.3)	(12.6)
Female (%)	32.9	24.7	42.4	37.5	45.2	40.5	36.6	34.7
Race/ethnicity (%)								
White	75.3	78.7	83.5	78.7	85.3	73.0	74.9	73.7
Black	9.7	7.0	6.6	7.9	5.0	15.5	11.2	15.7
Hispanic	8.3	7.7	5.8	7.6	5.3	7.2	7.8	6.8
Asian/Pacific Islander	2.8	3.2	1.6	2.6	1.6	1.6	2.8	1.3
Other race/ethnicity	4.0	3.5	2.5	3.3	2.8	2.6	3.3	2.5
Insurance coverage (%)								
Medicare	54.0	56.7	70.1	51.5	85.6	64.0	61.0	61.5
Medicaid	9.4	7.4	6.2	10.8	2.8	11.2	9.8	12.0
Private insurance	28.5	29.8	19.7	32.8	9.2	19.8	23.6	21.7
Other insurance	8.1	6.0	3.9	4.9	2.4	5.0	5.5	4.9
Income quartile (%)								
1st	29.7	27.9	28.5	23.9	21.9	31.8	27.7	32.9
2nd	27.4	27.5	28.6	25.8	25.6	27.5	27.0	28.2
3rd	23.9	24.7	24.5	25.9	26.5	23.2	24.9	22.9
4th	19.0	20.0	18.4	24.4	26.0	17.5	20.4	16.0
Patient location (%)								
Metro = 1 M population	50.3	47.0	45.3	51.7	52.1	50.2	50.8	48.4
Other metro (50–999 K)	30.9	33.1	34.3	31.6	31.2	31.5	31.6	32.3
Non‐metro	18.8	19.9	20.3	16.7	16.7	18.3	17.5	19.3

*Note:* Descriptive statistics for eight cardiovascular procedures, including weighted and unweighted case counts, demographics, elective admission status, APR‐DRG risk and severity, in‐hospital mortality, payer mix, income quartile, and urban–rural location. Standard deviation in parentheses for non‐percentile variables.

Abbreviations: APR‐DRG, all patient refined diagnosis related groups; CABG, coronary artery bypass grafting; CEA/CAS, carotid endarterectomy/carotid artery stenting; PCI, percutaneous coronary intervention; TAVR/TMVR, transcatheter aortic/mitral valve replacement.

Patients in the lowest income quartile comprised a substantial proportion of procedure volume, ranging from 21.9% for TAVR/TMVR to 32.9% for peripheral bypass. Mean age varied considerably by procedure, from 61.1 years for surgical valve replacement to 77.0 years for TAVR/TMVR. The proportion of elective admissions ranged from 9.2% for PCI to 77.7% for TAVR/TMVR, reflecting the different clinical contexts in which these procedures are performed.

### 3.2 Baseline Clinical Acuity by Socioeconomic Status

Patients from lower‐income areas consistently presented with higher baseline clinical acuity across all procedure types (Figure 1). A clear gradient in APR‐DRG risk of mortality scores by income quartile was evident, with patients in the lowest income quartile having meaningfully higher mean risk scores than those in the highest quartile across all procedure categories. This socioeconomic gradient in baseline clinical severity held for both risk of mortality (panel A) and severity of illness (panel B) scores.

Similar patterns emerged when examining clinical acuity by geographic location, though these differences were less pronounced than income‐based disparities. Non‐metropolitan patients generally presented with clinical acuity comparable to or slightly higher than metropolitan patients, though substantial variation existed across procedure types.

### 3.3 Overall Mortality Rates

In‐hospital mortality varied substantially across procedure types, ranging from 1.7% for CEA/CAS to 4.6% for non‐carotid endarterectomy and 4.6% for aneurysm repair (Table 1). These differences reflect both the varying complexity of procedures and the different patient populations undergoing each intervention.

### 3.4 Income Disparities in Mortality

Table 2 presents average marginal effects for in‐hospital mortality by income quartile and geographic location across the four model specifications. In the demographically adjusted model (Model 1), patients in the lowest income quartile had 0.55% points higher mortality compared to those in the highest quartile (95% CI 0.43–0.67). This disparity was attenuated but remained statistically significant after adjustment for clinical severity (Model 2: 0.24% points, 95% CI 0.12–0.36) and further reduced with hospital random effects (Model 3: 0.18, 95% CI 0.06–0.30).

**TABLE 2:** Average marginal effects on in‐hospital mortality by income quartile and patient location.

	(1)	(2)	(3)	(4)
Patient income quartile				
1st	0.55**	0.24**	0.18**	0.67*
	(0.43, 0.67)	(0.12, 0.36)	(0.06, 0.30)	(0.08, 1.26)
2nd	0.33**	0.16**	0.13*	0.50*
	(0.21, 0.45)	(0.04, 0.28)	(0.01, 0.25)	(0.01, 0.99)
3rd	0.20**	0.06	0.06	0.23
	(0.08, 0.32)	(−0.04, 0.16)	(−0.06, 0.18)	(−0.16, 0.62)
4th	[reference]	[reference]	[reference]	[reference]
Patient location setting				
Non‐metro	−0.18**	0.25**	0.14*	0.48
	(−0.30, −0.06)	(0.13, 0.37)	(0.02, 0.26)	(−0.01, 0.97)
Other metro (50 K‐999 K)	0.02	0.38**	0.29**	1.03**
	(−0.08, 0.12)	(0.28, 0.48)	(0.19, 0.39)	(0.30, 1.76)
Metro ≥ 1 M population	[reference]	[reference]	[reference]	[reference]
Demographic controls	X	X	X	X
APR‐DRG controls		X	X	X
Hospital RE			X	
Hospital FE				X
ICC			0.116	
Adjusted R‐squared	0.010	0.325		0.346
Observations	1,120,235	1,120,235	1,120,235	1,099,211

*Note:* Comparisons of four models: (1) logistic regression with demographics and year; (2) logistic regression adding All Patient Refined Diagnosis‐Related Groups (APR‐DRG) risk/severity scores; (3) multilevel logistic regression with hospital random effects (RE) intercepts; and (4) hospital fixed‐effects (FE) logistic regression. Models 1–3 incorporate NIS discharge weights; Model 4 does not incorporate survey weights due to methodological constraints of conditional fixed‐effects estimation. Models 1–3 use robust variance estimation clustered at the hospital level. Model 4 is a conditional fixed‐effects logistic model that conditions on hospital identity; the reported sample size is smaller because hospitals with no within‐hospital variation in outcomes are excluded from estimation. Reported statistics include average marginal effects (95% confidence intervals), adjusted *R* ^2^, intraclass correlation (ICC; for the multilevel model), and sample sizes. *p < 0.05, **p < 0.01.

Importantly, even in the hospital fixed effects model (Model 4), which compares patients treated at the same hospital, income disparities persisted. Patients from the lowest income quartile areas had 0.67% points higher mortality (95% CI 0.08–1.26), and those from the second quartile had 0.50% points higher mortality (95% CI 0.01–0.99) compared to the highest income quartile. The third income quartile showed a smaller, non‐significant elevation in risk (0.23% points, 95% CI −0.16 to 0.62).

**FIGURE 2:**
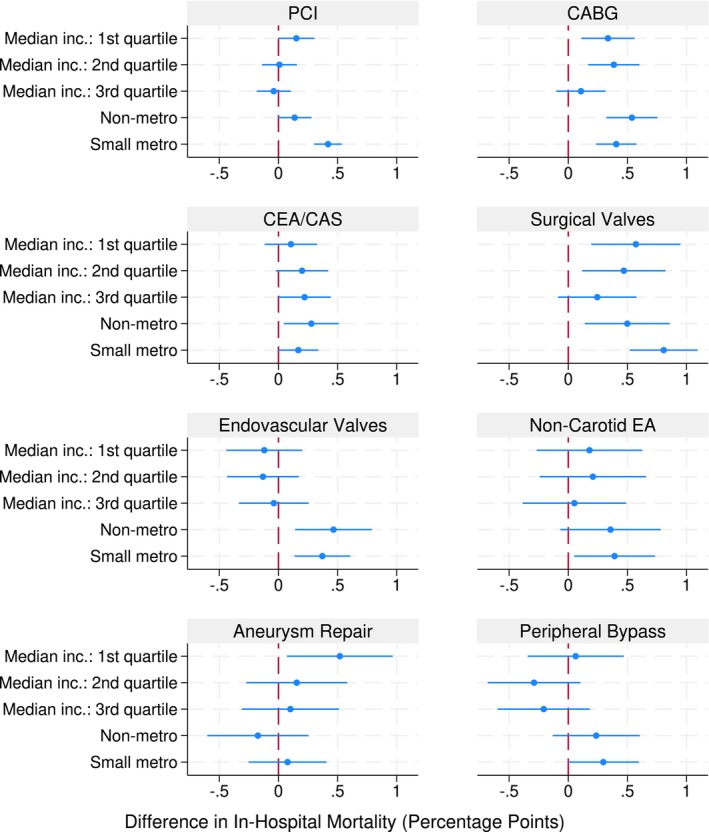
Adjusted differences on in‐hospital mortality by income quartile and patient location. Forest plots showing average marginal effect estimates (95% CI) for in‐hospital mortality by income quartile, estimated separately for each procedure using multivariable logistic regression (Model 2 specification). Models adjust for age, sex, race and ethnicity and their interactions, APR‐DRG risk of mortality and severity of illness scores, and calendar year. Reference categories: Income quartile 4 (highest) and large metropolitan areas (≥ 1 million population). APR‐DRG, all patient refined diagnosis related groups; CABG, coronary artery bypass grafting; CEA/CAS, carotid endarterectomy/stenting; PCI, percutaneous coronary intervention; TAVR/TMVR, transcatheter aortic/mitral valve replacement.

Figure 2 illustrates procedure‐specific average marginal effects from Model 2, demonstrating that income disparities were relatively consistent across all eight cardiovascular procedures, though the magnitude varied somewhat by procedure type.

### 3.5 Geographic Disparities in Mortality

Geographic disparities showed a complex pattern that depended critically on statistical adjustment. In the demographically adjusted model (Model 1), non‐metropolitan patients appeared to have lower mortality than those in large metropolitan areas (−0.18% points, 95% CI −0.30 to −0.06). However, this apparent advantage reversed after adjustment for clinical severity (Model 2), with non‐metropolitan patients showing 0.25% points higher mortality (95% CI 0.13–0.37).

The inclusion of hospital‐level factors (Models 3 and 4) attenuated but did not eliminate geographic disparities. In the hospital fixed effects model (Model 4), non‐metropolitan patients had 0.48% points higher mortality (95% CI −0.01 to 0.97), while patients from smaller metropolitan areas had 1.03% points higher mortality (95% CI 0.30–1.76) compared to those from large metropolitan areas.

### 3.6 Between‐Hospital Variation

The multilevel model (Model 3) yielded an intraclass correlation coefficient of 0.116, indicating that approximately 11.6% of the variance in mortality was attributable to between‐hospital differences. This ICC is not specific to income or geographic disparities but reflects overall variation in outcomes across hospitals beyond measured patient characteristics. The modest magnitude of the ICC suggests that while hospital‐level factors contribute to mortality differences, most variability occurs within hospitals, consistent with our finding that income‐based disparities persist among patients treated at the same facility.

The model fit statistics showed progressive improvement with each level of adjustment. The adjusted R‐squared increased from 0.010 in Model 1 to 0.325 in Model 2 and 0.346 in Model 4, indicating that clinical severity measures explained considerably more variance in mortality than demographic factors alone, but that hospital identity added relatively little additional explanatory power beyond clinical risk adjustment.

The similarity of estimates between Models 3 and 4 merits comment. Model 3 (random intercepts) and Model 4 (fixed effects) both adjust for hospital‐level heterogeneity, but do so differently: random effects estimate a distribution of hospital intercepts while fixed effects condition on hospital identity, eliminating all between‐hospital variation. When random and fixed effects yield similar estimates, it suggests that hospital‐level confounding is modest and that the random effects model adequately captures between‐hospital variation. The key comparison is Model 2 (pooled across hospitals) versus Model 4 (within‐hospital). Point estimates are similar or somewhat larger in Model 4, though confidence intervals widen considerably. Both income and geographic disparities persist within hospitals, indicating that differential access to high‐performing (lower‐mortality) institutions does not fully explain these gaps.

## 4 Discussion

In this nationally representative study of more than 1.1 million cardiovascular hospitalizations, we found persistent disparities in in‐hospital mortality by both income and geography. Three principal findings emerge. First, patients from lower‐income areas presented with greater illness severity and experienced higher mortality across all procedures, even after rigorous adjustment. Second, these income‐based disparities persisted among patients treated at the same hospital, suggesting mechanisms beyond differential access to high‐performing institutions. Third, income remained strongly associated with higher mortality within hospitals, while geographic differences persisted but were not consistently statistically significant.

The persistence of socioeconomic disparities within hospitals is particularly concerning. Patients from the lowest‐income location quartile faced 0.67% points higher mortality than those from the highest quartile, even when treated at the same facility. This pattern extends prior work demonstrating income gradients in survival after CABG, valve surgery, and myocardial infarction [16, 17, 31, 32, 33]. Several mechanisms may explain these findings. Lower‐income patients may present later in their disease course, with more advanced pathology not fully captured by risk‐adjustment models. They may also experience barriers to timely interventions or differences in perioperative management intensity, influenced by implicit bias or systemic inequities in clinical decision‐making [13, 14, 15]. Moreover, social determinants such as chronic stress, limited nutrition, and environmental exposures could exacerbate physiologic vulnerability to complications [34, 35, 36]. These factors point to within‐hospital inequities that go beyond what can be explained by hospital quality or referral patterns.

Geographic disparities showed a different pattern. In unadjusted models, nonmetropolitan patients appeared to have lower mortality, but this reversed after risk adjustment, consistent with referral and selection effects in rural health research [10, 12]. Once adjusted, patients from nonmetropolitan and smaller metropolitan areas had 0.25%–0.38% points higher mortality than those from large metropolitan regions. Fixed‐effects estimates were similar or larger but less precise, with the nonmetropolitan effect no longer statistically significant. Prior work points to hospital resources, procedural volume, and specialist availability as important drivers of these differences [37, 38, 39]. Policy approaches such as expanding tertiary care access, strengthening telemedicine networks, and improving emergency transport could help, but residual disparities suggest that travel distance, social support, and discharge coordination remain important barriers. Recent studies reinforce these patterns, showing that hospitals in more socioeconomically deprived regions perform fewer transcatheter and surgical valve procedures, with smaller differences for coronary operations. Emerging evidence also indicates that newer minimally invasive procedures are more unevenly distributed across regions, reflecting the concentration of advanced technologies and specialized staff, though these disparities appear to lessen as hospitals expand capacity and procedural experience grows [18, 20].

Income and geography likely interact in complex ways. Rural areas face disproportionately high poverty rates, and the intersection of socioeconomic disadvantage and geographic isolation may magnify disparities beyond the additive effects of each alone. Although our analyses treated these domains separately, future work should explicitly examine their joint effects as well as their interaction with race, ethnicity, and insurance status. This multidimensional perspective is critical to understanding the lived experience of disadvantage in cardiovascular care.

That patients from lower‐income locations consistently had higher APR‐DRG risk and severity scores points to upstream inequities. These patients face greater exposure to cardiovascular risk factors, reduced access to preventive care, and fewer opportunities for early disease, arriving for procedures with more advanced disease [40, 41, 42]. Addressing these upstream disparities will require extending efforts beyond hospitals to strengthen prevention, primary care, and community‐based interventions in disadvantaged populations.

The magnitude of the disparities observed in this study is consistent with prior U.S. evidence. The 0.67%‐point higher in‐hospital mortality among lowest‐income patients within the same hospital is comparable to—and in some cases exceeds—overall income gradients reported in studies that did not isolate within‐hospital effects, suggesting within‐hospital mechanisms account for a substantial share of previously documented disparities. Contemporary national estimates report income‐based differences of 0.5%–0.7% points and 50%–60% higher adjusted odds of death for CABG and valve surgery [43], with similar gradients for surgical aortic valve replacement but more attenuated differences for transcatheter procedures [44]. Geographic differences after risk adjustment—0.25%–0.38% points, or approximately 8%–12% relative increases given baseline mortality of 3%–4%—are comparable to Medicare and NIS studies of rural–urban disparities in acute myocardial infarction outcomes [11, 45]. That within‐hospital disparities match or exceed those reported elsewhere may suggest that care inequities within institutions warrant priority attention, though unmeasured factors may also contribute to these patterns.

Our study has several limitations. First, we relied on administrative data and APR‐DRG severity adjustment, which may not fully capture clinical complexity or unmeasured differences in disease severity between socioeconomic groups. Second, we used ZIP code‐level median income rather than individual‐level socioeconomic data, which introduces potential ecological fallacy and measurement error that likely attenuate observed associations. Third, we examined in‐hospital mortality only; socioeconomic and geographic disparities may be larger when considering 30‐day or longer‐term outcomes. Fourth, the NIS excludes federal hospitals and rehabilitation facilities, potentially underrepresenting vulnerable populations. Fifth, we could not assess surgeon or hospital procedural volume, which may mediate geographic disparities. Sixth, our analysis is conditional on procedure receipt; we cannot address disparities in access to procedures or appropriateness of patient selection. Seventh, we did not restrict our analysis to elective admissions; because urgent and emergent cases have higher baseline mortality risk, the inclusion of non‐elective admissions may introduce confounding if admission urgency differs systematically by income or geography, potentially affecting the magnitude of observed disparities. Eighth, while hospital fixed effects control for all time‐invariant hospital characteristics, they cannot account for temporal changes in hospital quality or capacity. Additionally, Model 4 was estimated without survey weights, and comparisons with the weighted models should therefore be interpreted with some caution, notwithstanding the consistency of point estimates across specifications. Finally, residual confounding from unmeasured patient factors (e.g., social support, health literacy, and medication adherence) may influence our estimates.

Why income disparities persist within the same hospital is not well understood. Our risk adjustment accounts for measured clinical severity, yet patients from lower‐income locations still face higher mortality. This could reflect unmeasured disease severity, differences in treatment decisions or timing, variable physiologic reserve, or other factors not captured in administrative data. Determining which mechanisms matter most requires studies linking individual‐level social and clinical data—something administrative databases cannot provide. Without this knowledge, interventions remain speculative. In this context, place‐based policy efforts such as the Rural Health Transformation Program may help mitigate geographic disparities through investments in rural hospital capacity, but are unlikely to fully address income‐related mortality differences that persist within hospitals. Meanwhile, performance‐based payment programs continue to penalize hospitals serving vulnerable populations, potentially widening the very disparities they aim to reduce [10, 26, 27, 46].

## 5 Conclusions

Significant socioeconomic and geographic disparities in mortality following major cardiovascular procedures persist despite risk adjustment for clinical severity. Income‐based disparities remain pronounced within hospitals, suggesting that mechanisms beyond differential access to high‐quality facilities contribute to these inequities. Geographic disparities, by contrast, are estimated with greater uncertainty in within‐hospital analyses and are not consistently statistically significant, indicating a larger role for hospital‐level factors. Multilevel interventions targeting both upstream social determinants and within‐hospital care processes are needed to reduce these persistent inequities.

## Funding

The authors have nothing to report.

## Conflicts of Interest

The authors declare no conflicts of interest.

## Data Availability

The data that support the findings of this study are available from Agency for Healthcare Research and Quality. Restrictions apply to the availability of these data, which were used under license for this study. Data are available from the author(s) with the permission of Agency for Healthcare Research and Quality.
